# A Step Forward in Precision Medicine on “One Belt One Road”

**DOI:** 10.1016/j.gpb.2017.08.001

**Published:** 2017-08-12

**Authors:** Jun Yu

**Affiliations:** CAS Key Laboratory of Genome Sciences and Information, Chinese Academy of Sciences, Beijing 100101, China

The story started nearly a year ago. On October 13 in 2016, a *News & Views* article entitled “Precision medicine: what challenges are we facing?”, written by a group of 21 bioinformaticians in China, who are actively involved in the relevant research, was released online in *Genomics Proteomics Bioinformatics* [1]. A month later,an invitation arrived from a Thai colleague to one of the authors, Professor Yu Xue. The Thai colleague is Professor Somchai Bovornkitti, who is a member of the Royal Society of Thailand (RST) and serves as the chairman of the Health Forum, Academy of Science, RST. He wrote to express intention to invite a representative from the group of authors to present in a forthcoming Association of Southeast Asian Nations (ASEAN) meeting on precision medicine (PM) in June 2017. Professor Xue did ask around for a single volunteer; instead, he had gotten a dozen.

Of course, the story did not end at this point. As a matter of fact, the meeting was upgraded to be the Sino-ASEAN Conference on Precision Medicine focusing on the current state of PM in the Sino-ASEAN context. The conference was held during June 17–18, 2017 in Burapha University, Chon Buri, Thailand. Eight other outstanding young Chinese scientists, in addition to myself – an old timer working on genomics and bioinformatics, showed up at the conference and delivered excellent talks on their successful career achievements covering diverse topics in omics and bioinformatics, which include, but not limited to, application of posttranscriptional modifications in cancer, big data in genomics and PM, association studies in psychological disorders, DNA methylation and variation detection/annotations in cancers, and population diversity in China. In addition, 25 speakers from Thailand and other ASEAN countries also presented their talks that cover even more topics from PM application in various diseases, therapies used for many common diseases, all the way to medicinal plants, stem cells, and longevity.

The major theme of the conference became promoting multi-disciplinary collaborations between basic biomedical and clinical research fields, as all conference attendees strongly agreed. PM projects unite large-scale biology, such as genomics, proteomics, and bioinformatics, and medicine, to promote technological development in the meantime.

What’s more, a sequel of the conference was announced at the meeting, which will be held somewhere in China in 2018. Our Chinese colleagues will have time to know and entertain their Thai hosts and new friends from ASEAN. There will definitely be another step toward to connect scientists along “the One Belt One Road”, also known as “the Silk Road Economic Belt and the 21st-century Maritime Silk Road (or Belt and Road Initiative, BRI), an economic and political development strategy proposed by China's paramount leader President Xi Jinping, which focuses on connectivity and cooperation among Eurasian countries.
